# Reports on Hospitals of the United Kingdom

**Published:** 1914-03-28

**Authors:** Henry Burdett


### REPORTS ON

### Hospitals of the United Kingdom

By SIR HENRY BURDETT, K.C.B., K.C.V.O.

SERIES III.

### HORSHAM COTTAGE HOSPITAL.

THIS hospital, established in 1892 and enlarged in 1908 and 1910, contains twelve beds, and the pressure on these beds is reported to be continually increasing. There is, therefore, a demand to extend one side of the hospital by building out at the back on the plan followed on the other side. We hope this idea will not be adopted, because the distance between the two wings would then be too small, and, on hygienic and other grounds, a better scheme should be obtained before any extension of the hospital is sanctioned. We make a strong point of this, because most excellent surgery is done, and no extensions should be undertaken without the fullest apprehension of their effect, and under expert advice only.

The patients amount to some 180 annually at present, and the average number of beds constantly occupied is nine. The hospital seems to have been erected without any carefully-thought-out plan, and is the first institution of the kind in which we have found the entrance to the committee room through the kitchen. Of course, this room, which is one of the best in the hospital, is used for all kinds of other purposes, but it is matter for wonder why such a fine apartment was ever erected in the position which this committee room occupies.

We had the pleasure of meeting the senior member of the staff, Mr. M. H. Vernon, and were glad to notice his enthusiasm and keen interest in regard to the best interests of the sick and of the Cottage Hospital. This hospital has a mortuary, which we are pleased to say exhibits a keen apprehension on the part of the authorities of the respect which should be shown to the dead and the fullest consideration for the feelings of the friends. We would suggest that a few annual reports should be kept at the hospital, so that anyone interested might have an opportunity of gaining information about its work. A report enables a visitor to learn something about a hospital and its work before he proceeds to see the buildings.

### THE ELIOT COTTAGE HOSPITAL, HAYWARD'S HEATH.

#### King Edward VII. Memorial.

THIS hospital was started in 1906, rebuilt in 1911 on a new site, and opened in 1912. It contains twelve beds, and has been equipped and fitted up with a completeness seldom or never attempted in the early days of an institution of this type. Early in 1910, the matron, Miss Mary Barrett, who was trained at the Middlesex Hospital, wrote to us requesting that we should pay a visit to the hospital, which then consisted of two houses, with accommodation for twelve patients. At that time, owing to the great increase in the work, the committee were considering the advisability of having plans drawn up for rebuilding the hospital on a new site. It was the matron's wish that we should co-operate in the preparation of the plans. Had we been able to do so, we should certainly have seen that the wards were given thorough cross-ventilation.

The site of the hospital is good, and there are extensive views from the back of it (which faces north). That is the reason, we presume, the architect has placed the wards facing the road with a southern aspect. The whole of the hospital was in the most perfect order and presented an attractive and home-like appearance. The nursing and domestic staff of few hospitals, indeed, have accommodation and comforts equal to those provided at the Eliot Cottage Hospital, Hayward's Heath. The hospital, as it stands, is indeed a memorial of good and conscientious work among the sick by an intelligent and enthusiastic matron, whose years of devoted work in the district have borne such excellent fruit.

The hospital has, at present, only a temporary shed in use as a mortuary, which has been made as suitable as possible in the circumstances. We hope that some man or woman, possessed of imagination and a keen desire to make this Cottage Hospital an object-lesson in hygiene and the care of the sick, which every such institution ought to be, will take up the question of the mortuary and co-operate to secure a model unit for this section of this institution, with a *post-mortem* room and a view-room or chapel. We congratulate the committee, the matron, and everybody who has had a hand in providing Hayward's Heath and the surrounding district with this pleasant and efficient hospital, which it was a great pleasure to visit. We have received no report or statement of accounts of this hospital. It is good business to provide that copies of the annual reports shall be kept at every hospital, so that visitors may readily obtain one and so become interested in the work.

